# Down‐regulation of PDGFRβ suppresses invasion and migration in osteosarcoma cells by influencing epithelial–mesenchymal transition

**DOI:** 10.1002/2211-5463.12915

**Published:** 2020-08-03

**Authors:** Sining Xing, Changdong Wang, Huying Tang, Jiaqi Guo, Xing Liu, Faping Yi, Geli Liu, Xiangmei Wu

**Affiliations:** ^1^ Department of Physiology Chongqing Medical University Chongqing China; ^2^ Molecular Medicine and Cancer Research Center Chongqing Medical University Chongqing China; ^3^ Department of Biochemistry and Molecular Biology Chongqing Medical University Chongqing China; ^4^ Department of Pediatric Urology Chongqing Children’s Hospital Chongqing Medical University Chongqing China

**Keywords:** epithelial–mesenchymal transition, invasion, migration, osteosarcoma, PDGFRβ

## Abstract

Osteosarcoma (OS) is the most common malignant bone tumor primarily influencing children and adults. Approximately one‐fifth of patients have micrometastasis in the lungs when OS is diagnosed. Platelet‐derived growth factor receptor (PDGFR) beta (PDGFRβ) is a subtype of PDGFR. PDGFRβ has been noted to be highly expressed in OS cell lines and patient specimens, and is associated with metastasis and poor prognosis of OS. However, mechanistic insights into the exact role of PDGFRβ in OS pathogenesis and development are still lacking. Here we assessed the effects of PDGFRβ on invasive and migratory abilities, such as the epithelial–mesenchymal transition and phosphatidylinositol 3‐kinase (PI3K), Akt and mammalian target of rapamycin (mTOR) pathways in HOS cells. Depleting PDGFRβ resulted in reduced migration of HOS cells in the small interfering RNA duplexes specific for the PDGFRβ group compared with the mock and scramble‐treated groups in Transwell invasion assays. Using wound‐healing assays, we demonstrate the rate of wound healing in the PDGF‐BB‐stimulated group was higher compared with the mock‐treated group. Western blot showed that down‐regulation of PDGFRβ decreased the expression of stromal phenotype markers and phosphorylation pathway proteins (PI3K, AKT and mTOR), but the epithelial phenotype marker was increased in HOS cells. Treating HOS cells with PDGF‐BB revealed a treatment time‐dependent increase of phosphorylated, but not total, PI3K, AKT and mTOR. Taken together, we suggest that PDGFRβ plays an important role in OS invasion, migration and epithelial–mesenchymal transition by influencing the PI3K, Akt and mTOR pathways, hence highlighting PDGFRβ as a potential therapeutic target for OS.

## Graphical abstract

Platelet‐derived growth factor receptor beta belongs to the tyrosine kinase family. Platelet‐derived growth factor receptor beta binds to its specific ligand and induces autophosphorylation to activate the downstream phosphatidylinositol 3‐kinase/AKT/mammalian target of rapamycin pathway. The activated pathway molecules then influence the epithelial–mesenchymal transition process by regulating the expression of epithelial–mesenchymal transition‐related proteins, and ultimately regulating tumor metastasis.

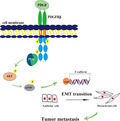

**Abbreviations**
CCK‐8: Cell Counting Kit‐8
EMT: epithelial–mesenchymal transition
mTOR: mammalian target of rapamycin
OS: osteosarcoma
p: phosphorylated
PDGFR: platelet‐derived growth factor receptor
PDGFRβ: platelet‐derived growth factor receptor beta
PI3K: phosphatidylinositol 3‐kinase
qRT‐PCR: quantitative RT‐PCR
RTK: receptor tyrosine kinase
si‐NC: nontargeting control siRNA
si‐PDGFRβ: small interfering RNA duplexes specific for PDGFRβ
siRNA: small interfering RNA

As a cancerous tumor that originates from bone, osteosarcoma (OS) is the most frequent histological type of primary bone cancer and is becoming the second leading cause of cancer‐related deaths in children and adolescents [1]. It accounts for ~20% of all primary bone cancers and 2.4% of all malignancies, with high mortality and morbidity in children [2]. Tumor metastasis, especially lung metastasis, is the main reason for the death of patients with OS [3]. Approximately one‐fifth of the patients have micrometastasis in the lungs when OS is diagnosed. Surgery is an important way to treat this disease, but the subsequent complications may result in more serious consequences [4]. A variety of agents have been investigated for the treatment of OS in clinical trials. Although several studies focus on the molecular mechanism of OS, the specific mechanism remains unclear. Further research on OS pathogenesis may provide new ideas for the treatment of OS. With the discovery of an increasing number of molecular mechanisms that can mediate the invasion and metastasis of OS, epithelial–mesenchymal transition (EMT) has aroused interest [5, 6, 7]. EMT is one of the transformations by which tumor cells can acquire the ability to migrate and is an important process in tumor cell infiltration and metastasis [8, 9]. It has been reported that EMT is highly correlated with the invasive and metastatic performances of many types of tumor cells [10, 11] and especially promotes the metastasis of epithelial neoplasms [12]. EMT also plays a pivotal position in primary and secondary metastases in OS [13].

Receptor tyrosine kinases (RTKs) are key players in the regulation of numerous fundamental cellular processes, such as growth, migration and apoptosis, and are involved in tumorigenesis, disease progression and metastatic spread of numerous human cancers [14, 15, 16, 17]. Platelet‐derived growth factor receptor (PDGFR) belongs to the family of type III tyrosine protein kinases, and PDGFR beta (PDGFRβ) is an important subtype of PDGFR. PDGFRβ has been noted to be highly expressed in OS cell lines and patient specimens [18], and is associated with metastasis and poor prognosis of OS. Activating the PDGFRβ‐Akt signaling pathway can increase the growth of OS cells [19] and further deepen the malignancy of OS. However, mechanistic insights into the exact role of PDGFRβ in OS pathogenesis and development are still awaited.

In this study, we focused on the specific role that PDGFRβ played in invasive and migratory abilities of HOS cells and the possible mechanism. At first, we screened out the best inhibition target for PDGFRβ and the best concentration of the highest‐affinity ligand for PDGFRβ, PDGF‐BB. In addition, we investigated the role of PDGFRβ in invasive and migratory abilities, and explored EMT and the molecular pathway phosphatidylinositol 3‐kinase (PI3K)/Akt/mammalian target of rapamycin (mTOR) of HOS cells.

## Materials and methods

### Cell line and culture

The human OS cell line HOS was purchased from Shanghai Cell Bank of Chinese Academy of Sciences (Shanghai, China). The cells were cultured in Dulbecco’s modified Eagle’s medium containing 10% FBS at 37 °C in a humidified 5% CO_2_ incubator.

### RNA interference

Small interfering RNA (siRNA) duplexes specific for PDGFRβ (si‐PDGFRβ) and nontargeting control siRNA (si‐NC) duplexes were obtained from GenePharma Corporation (Shanghai, China). Lipofectamine RNAiMAX (Invitrogen, Shanghai, China) was used for transfection according to the manufacturer's instructions. After transfection for 24 h, cells were collected for the subsequent experiments. siRNA sequences are as follows: si‐NC sense, 5′‐UUCUCCGAACGUGUCACGUTT‐3′; antisense, 5′‐ACGUGACACGUUCGGAGAATT‐3′; si‐PDGFRβ1,forward:5′‐GUGAGAAGCAAGCCCUUAUTT‐3′, reverse: 5′‐AUAAGGGCUUGCUUCUCACTT‐3′; si‐PDGFRβ2, forward: 5′‐CUCCAGUGCUAAGCUACAUTT‐3′, reverse: 5′‐AUGUAGCUUAGCACUGGAGTT‐3′; si‐PDGFRβ3, forward: 5′‐GAGGGUGACAACGACUAUATT‐3′, reverse: 5′‐UAUAGUCGUUGUCACCCUCTT‐3′.

### Cell Counting Kit‐8 array

Cells were plated and directly cultured with different concentrations of PDGF‐BB (1, 3, 10, 15, 30 and 50 μm). The cells were incubated at 37 °C under 5% CO_2_ and then incubated for an additional 1 h with 10 μL Cell Counting Kit‐8 (CCK‐8; Boster, Wuhan, China) for 0, 1, 2, 3, 4 and 5 days. The absorbance at 450 nm was measured using a microplate reader.

### RNA extraction and quantitative RT‐PCR

Total RNA was extracted from cell lines using TRIzol Reagent (Invitrogen, Carlsbad, CA, USA) according to the manufacturer’s protocol. cDNA synthesis was performed using a Prime Script RT Reagent Kit with gDNA Eraser (Takara, Dalian, China). Real‐time PCR analyses were performed with SYBR Green PCR Master Mix (Takara). The sequences of specific RNA primers for PDGFRβ and GAPDH were as follows: PDGFRβ, sense, 5′‐TGGCTAGCTCAGGGCTTCAG‐3′ and reverse, 5′‐TCTCCTTGCCAAGCTTCCTTC‐3′; GAPDH, sense, 5′‐GAAGGTGAAGGTCGGAGTC‐3′ and reverse, 5′‐GAAGATGGTGATGGGATTTC‐3′.

The quantitative RT‐PCR (qRT‐PCR) and data collection were performed on an Applied Biosystems 7500 Real‐Time PCR System (Foster City, CA, USA). Relative mRNA expression levels were calculated using the $2^{‐\Delta \Delta C_{t}}$ method. Each experiment was performed in triplicate.

### Western blot

Cellular proteins were extracted using radioimmunoprecipitation assay protein extraction reagent (Beyotime, Shanghai, China) according to the manufacturer’s instructions. The protein concentration in the cell supernatants was measured with a Bicinchoninic Acid Protein Assay Kit (Beyotime, Shanghai, China). Samples were resolved by 8–10% SDS/PAGE. The proteins were then transferred to a poly(vinylidene difluoride) membrane (Solarbio, Beijing, China). For western blots, the membranes were incubated at room temperature for 2 h in 5% nonfat dry milk solution in Tris‐buffered saline–Tween 20 and subsequently incubated with primary polyclonal antibodies (anti‐PDGFRβ, anti‐AKT, anti‐p‐AKT473 and anti‐β‐actin [Abcam, Cambridge，UK]; anti‐E‐cadherin, anti‐alpha smooth muscle actin (α‐SMA), anti‐Vimentin and anti‐GAPDH [Zhengneng, Chengdu, China]) at 4 °C overnight. A peroxidase‐linked secondary antibody (Zhongshan Golden Bridge, Beijing, China) was then incubated with the poly(vinylidene difluoride) membranes for 1 h, and enhanced chemiluminescence was used to visualize the bands. β‐Actin served as loading controls.

### Immunofluorescence

The slides were placed in a 24‐well plate, and the HOS cells were transfected for 48 h with 5 × 10^4^ cells per well. Subsequently, the slides climbing the cells were washed three times with PBS every 3 min, fixed with methyl alcohol for 15 min at 37 °C, washed again with PBS and blocked with blocking buffer (normal goat serum) for 60 min at 37 °C. After removal of excess blocking buffer, the slides were incubated with primary polyclonal antibodies (E‐cadherin, 1 : 50 [Zhengneng, Chengdu, China]; vimentin, 1 : 50 [Zhengneng, Chengdu, China]) at 4 °C overnight, washed with PBS and incubated with fluorescent secondary antibody at 37 °C for 1 h in the dark. Finally, the slides were washed with PBS and incubated with DAPI (4',6‐diamidino‐2‐phenylindole) in the dark for 5 min to stain nuclear. Cells were imaged using a fluorescent microscope at 400× magnification.

### Wound‐healing assays and Transwell assays

Transfected and PDGF‐BB‐stimulated cells were cultured in six‐well plates until confluent and then scratched to create wounds using 10‐µL sterile plastic micropipette tips. Cells were further cultured with serum‐free medium. At 0, 24 and 48 h after scratch, photographs were taken with a microscope at 100× magnification.

Transwell migration assays were performed to detect cell invasion and migration. Transwell chamber was used for migration assay. A total of 1 × 10^4^ 48‐h siRNA‐transfected cells and 48‐h PDGF‐BB‐stimulated cells were seeded separately in the upper chamber (8‐μm pore size; Corning Life Sciences，New York，USA) with 200 μL serum‐free medium, whereas the lower chamber was filled with 600 μL medium supplemented with 10% FBS. After 24 h, the cells were fixed, stained and counted. Migrating cells were counted using a microscope at 100× magnification. A total of 2 × 10^4^ cells seeded in the Transwell chamber inserts with Matrigel were used for invasion assay. Other conditions are the same as the migration assay. The invasion cells were counted using a microscope at 200× magnification.

### Statistical analysis

spss 13.0 package (SPSS Inc., Chicago, IL, USA) was used for the data analysis. All experiments were repeated at least three times. The data are expressed as the mean ± SD, and *P < *0.05 was considered statistically significant. A repeated‐measures variance test was used to analyze time point data.

## Results

### Screening the best inhibition target for PDGFRβ and the best concentration of PDGF‐BB

**Fig. 1:**
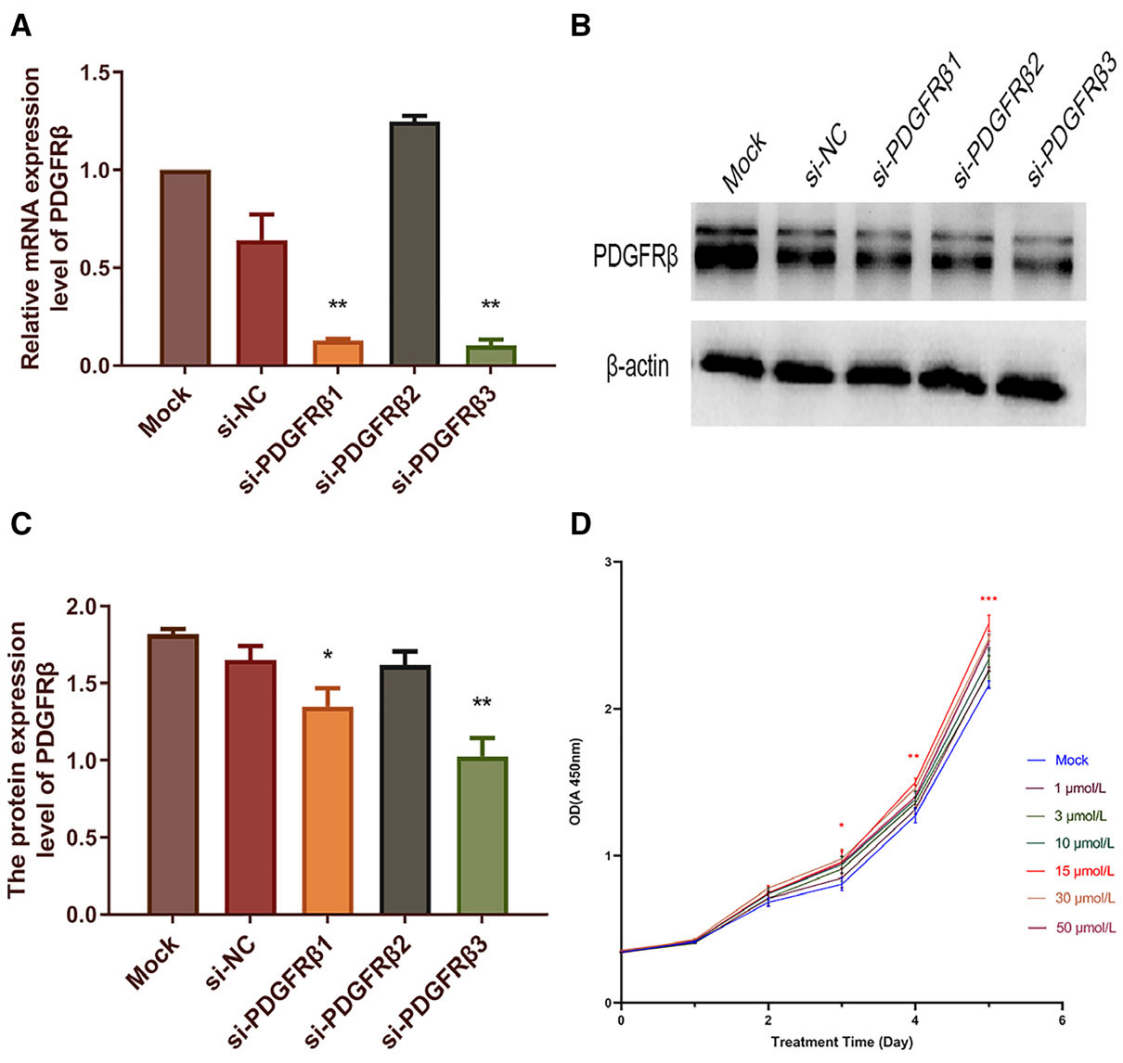
Screening of the best inhibition target for PDGFRβ and the best concentration of PDGF‐BB. The HOS cells were separately transfected with three siRNA‐PDGFRβ sequences (1–3) respectively to down‐regulate the expression of PDGFRβ. After transfection for 48 h, the relative expression of PDGFRβ mRNA in HOS cells was examined by qRT‐PCR (A), and GAPDH served as an internal control. After transfection for 72 h, the expression of PDGFRβ on protein level was examined by western blot assay, and protein bands were quantified (B, C). Densitometric analysis of each protein level was calculated from the average of three experiments, and β‐actin was used as an internal control. After PDGF‐BB was stimulated, cell viability of HOS cells was determined by CCK‐8 assay (D). Differences between multiple groups were compared with the one‐way ANOVA, followed by Newman–Keuls posttest. Data represent the mean ± SD of three independent experiments. *P < 0.05, **P < 0.01, ***P < 0.001.

We screened a small interference sequence with the strongest interference efficiency based on the results of mRNA and protein analysis. The results of qRT‐PCR (Fig. 1A) and western blot (Fig. 1B,C) revealed that PDGFRβ was obviously down‐regulated at both the mRNA and the protein levels after transfection with si‐PDGFRβ3. Therefore, the sequence si‐PDGFRβ3 was chosen to down‐regulate the expression of PDGFRβ for the following assays. The group transfected with si‐PDGFRβ3 was named si‐PDGFRβ. The group transfected with the scramble sequence was used as the si‐NC group. The CCK‐8 assay was used to detect the proliferation of OS cells after PDGF‐BB stimulation to select the best concentration according to the results (Fig. 1D). According to the growth curve results of different concentrations of PDGF‐BB (1, 3, 10, 15, 30 and 50 μm), we found the cell proliferation was most pronounced when the concentration of PDGF‐BB was 15 μm. Therefore, 15 μm was used for subsequent experiment.

### PDGFRβ influenced the invasion and migration of HOS cells

**Fig. 2:**
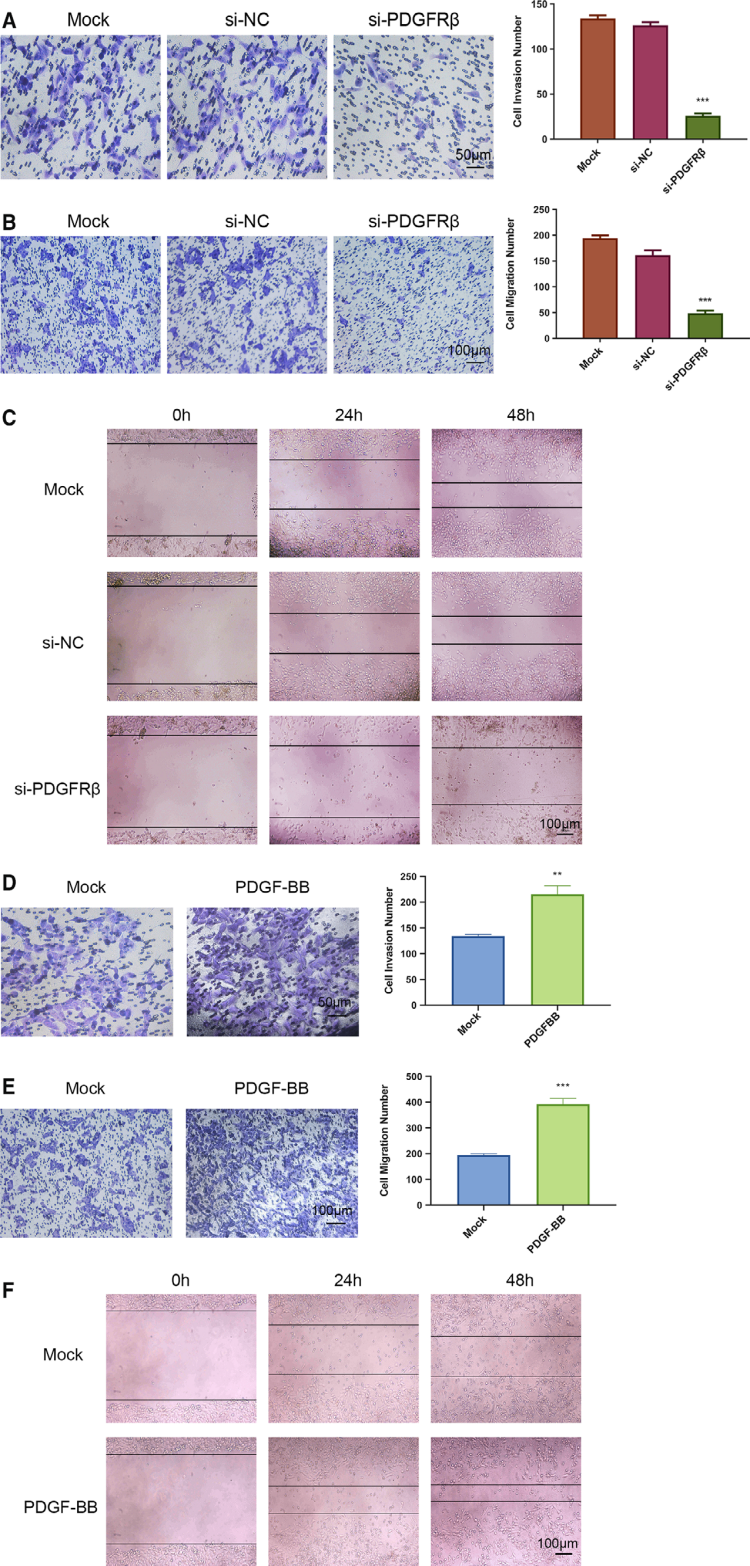
PDGFRβ influenced the invasion and migration of HOS cells. After PDGFRβ was down‐regulated, the invasion ability of HOS cells was detected by Transwell invasion assay (A). Scale bar: 50 μm. The migration ability of HOS cells was detected by Transwell migration assay (B) and wound‐healing assay (C). Scale bar: 100 μm. After PDGF‐BB stimulation, the invasion ability of HOS cells was detected by Transwell invasion assay (D). Scale bar: 50 μm. The migration ability of HOS cells was detected by Transwell migration assay (E) and wound‐healing assay (F). Scale bar: 100 μm. Differences between two groups were compared using Student’s *t*‐test (versus Mock group). Data represent the mean ± SD of three separate experiments. **P < 0.01, ***P < 0.001.

Transwell invasion assay was used to determine the invasion ability of HOS cells. Transwell migration assay and wound‐healing assay were used to determine the migration of HOS cells. According to our results, after down‐regulating the expression of PDGFRβ, the number of HOS cells that successfully passed through Matrigel was significantly reduced (Fig. 2A). As experimental results related to the detection of migration ability showed, down‐regulation of PDGFRβ reduced the migration of HOS cells; the average counts of migrating cells in the si‐PDGFRβ group were much lower than those of the si‐NC and Mock groups (Fig. 2B). The wound area in the si‐PDGFRβ group was larger than that in the other two groups (Fig. 2C). Whereas after stimulation with PDGF‐BB for 48 h, the results were just the opposite. The numbers of invaded (Fig. 2D) and migrated (Fig. 2E) cells were significantly increased. The wound‐healing assay (Fig. 2F) after scratching for 24 h showed that compared with the Mock group, the rate of wound healing in the PDGF‐BB‐stimulated group was higher, demonstrating better wound‐healing ability. In summary, these results showed that PDGFRβ is related to the invasion and migration ability of HOS cells.

### Down‐regulation of PDGFRβ influenced the expression of EMT‐related genes in HOS cells

**Fig. 3:**
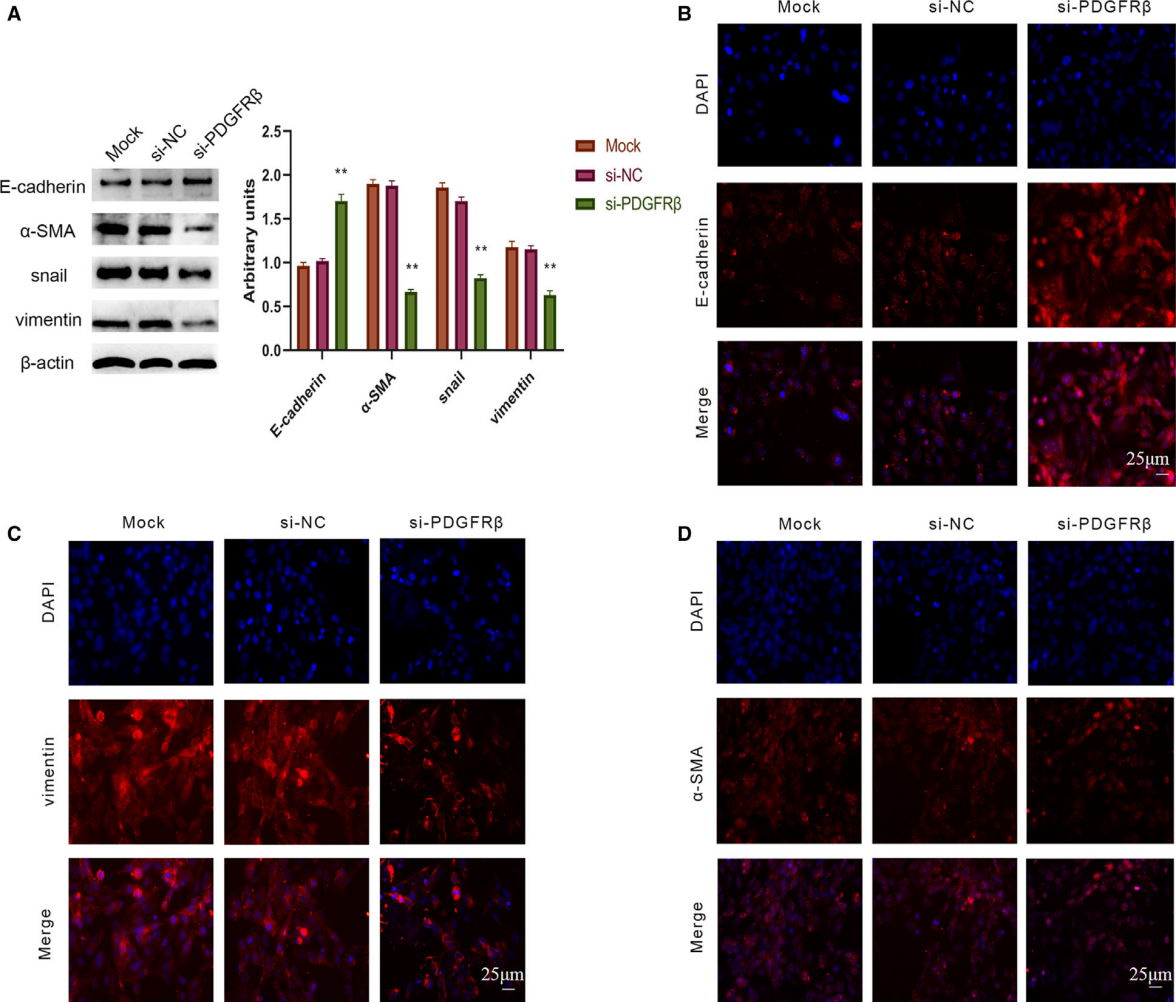
Down‐regulation of PDGFRβ influenced the expression of EMT‐related genes in HOS cells. After treatment with corresponding siRNA for 48 h, EMT‐related genes in HOS cells, including E‐cadherin, vimentin, Snail and α‐SMA, were measured by western blot (A). Densitometric analysis of each protein level was calculated from the average of three experiments, and β‐actin served as an internal control. The expressions of E‐cadherin (B), vimentin (C) and α‐SMA (D) were shown by immunofluorescence staining. Scale bars: 25 μm. Differences between multiple groups were compared with one‐way ANOVA, followed by Newman–Keuls posttest. Data represent the mean ± SD of three separate experiments. **P < 0.01.

Furthermore, we explored the role of PDGFRβ on EMT‐related genes expression in HOS cells. The protein expression of EMT‐related genes of HOS cells after transfection for 72 h was analyzed (Fig. 3A). We found that down‐regulation of PDGFRβ reduced the expression of vimentin, Snail and α‐SMA. However, the expression of E‐cadherin was up‐regulated. Subsequently, we detected the expression of E‐cadherin (Fig. 3B), vimentin (Fig. 3C) and α‐SMA (Fig. 3D) in HOS cells by immunofluorescence assay. The expression of E‐cadherin was increased in the HOS cells after 48 h of treatment with si‐PDGFRβ, and the expressions of vimentin and α‐SMA were decreased compared with that in the controls, which corresponds to western blot assay result.

### PDGFRβ regulated the PI3K/AKT/mTOR signal pathway

**Fig. 4:**
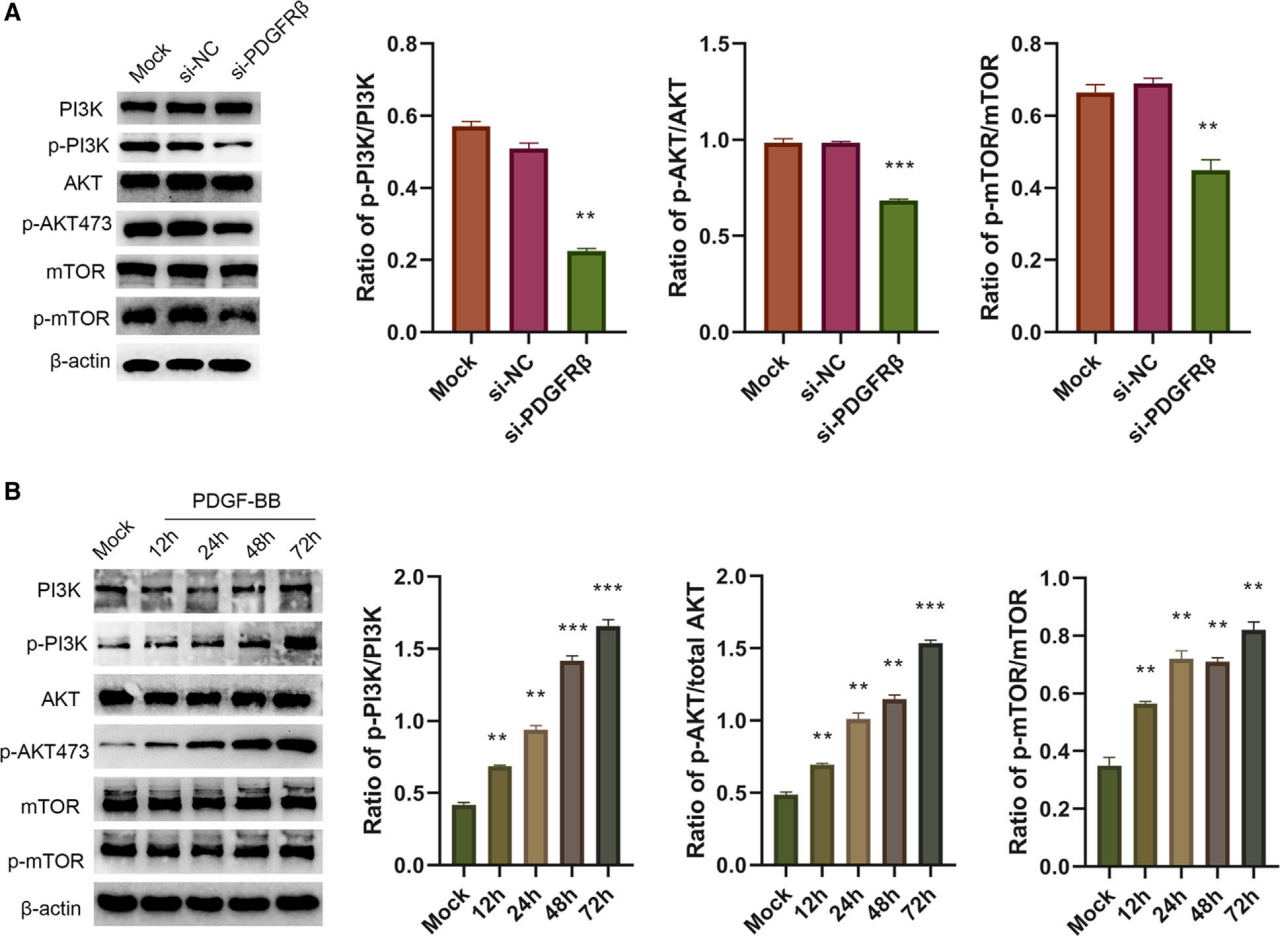
PDGFRβ regulated the PI3K/AKT/mTOR signal pathway. HOS cells were transfected with corresponding siRNA; then total PI3K, AKT, mTOR and their phosphorylation levels were measured by western blot assay (A). HOS cells were stimulated by PDGF‐BB for 0, 12, 24, 48 and 72 h, then subjected to western blot assay (B). Differences between multiple groups were compared with one‐way ANOVA, followed by Newman–Keuls posttest. Data represent the mean ± SD of three independent experiments. **P < 0.01, ***P < 0.001.

PI3K/AKT/mTOR is an important signal pathway for tumorigenesis and development. We analyzed by western blot assay several key molecules relevant with this pathway, such as PI3K, AKT and mTOR, as well as the changes in their phosphorylation levels. The results showed that down‐regulation of PDGFRβ inhibited the phosphorylation levels of PI3K, AKT and mTOR, whereas no obvious changes were observed in the total PI3K, AKT and mTOR levels (Fig. 4A). The HOS cells were stimulated with PDGF‐BB for 0, 12, 24, 48 and 72 h, then subjected to western blot assay (Fig. 4B). The results showed that the expressions of phosphorylated (p)‐PI3K, p‐AKT473 and p‐mTOR were increased, whereas total PI3K, AKT and mTOR were approximately unchanged. The longer the stimulation time, the higher was the phosphorylation level expression.

## Discussion

PDGFR has been shown to be highly expressed in numerous tumors and can mediate phosphorylation signal transduction to promote tumor cell growth, tumor stromal cell proliferation and tumor angiogenesis. PDGFRβ is an important subtype of PDGFR. After binding to the corresponding ligands, PDGFRβ can lead to OS cell proliferation through downstream signal molecules, such as AKT, ERK1/2 (extracellular regulated protein kinases 1/2) and STAT5 (signal transducers and activators of transcription 5).

OS remains one of the most prevalent malignancies and has poor overall survival rates [20]. Metastasis has been found in 85% of patients with OS, and the most common site of metastasis is the lung [6, 21]. Metastasis is the most common cause of OS death. Currently, metastasis of OS has become the main difficulty in OS treatment. Finding and delving into a metastasis mechanism should receive more attention. Our studies have demonstrated that the ability of cells to invade and migrate was reduced after down‐regulation of PDGFRβ. After stimulation by PDGF‐BB, the invasion and migration of HOS cells were enhanced. This indicates that PDGFRβ does have an effect on the invasion and migration of HOS cells.

In recent years, many studies have suggested that EMT is an important mechanism for metastasis of tumors. EMT transforms cell change from epithelial cell phenotype to mesenchymal‐like phenotype, can extensively remodel the cytoskeleton and can improve the ability to migrate [22]. Epithelial cells lose cell polarity; the expression of epithelial cell markers, such as keratin and E‐cadherin, is decreased; and the expression of cell adhesion molecules is reduced or lost, so that the cell–cell adhesion is weakened. The expression of stromal cell markers destroys the cell basement membrane and extracellular matrix, and ultimately promotes the invasion and migration of cancer cells. Down‐regulation or deletion of E‐cadherin expression is a typical manifestation of EMT [23]. Our experimental studies show that the expression of epithelial phenotype marker E‐cadherin was increased after PDGFRβ was down‐regulated. Vimentin is currently used as an important marker protein for mesenchymal cells, and its positive or increased expression is considered to be an indicator of cancer cell invasion phenotype [24]. As the first member of the Snail family [25], Snail plays an important role in EMT. Studies have shown that Snail directly binds to the promoter of E‐cadherin, down‐regulates the expression of E‐cadherin, reduces tumor adhesion, disrupts normal tissue morphology and promotes tumor invasion [26]. In addition, knockdown of Snail1 significantly attenuated the apoptosis and invasiveness of OS cells [27]. Furthermore, α‐SMA as a mesenchymal marker also plays a very important role in the EMT process. In our study, after PDGFRβ was inhibited, the expressions of stromal phenotype markers vimentin and α‐SMA were decreased, as well as the transcription factor Snail. The results of immunofluorescence were consistent with those of western blot. All of these results showed that PDGFRβ may influence invasion and migration of HOS cells by EMT. However, OS is a stromal tumor, and specific function of EMT in it still needs further study.

PDGFRβ can regulate multiple signaling pathways, such as the PI3K/AKT pathway, Ras/MAPK pathway and STAT pathway, of which PI3K/Akt/mTOR plays a crucial role in tumorigenesis and progression [28, 29], participates in angiogenesis and regulates changes in the cell cycle [30]. In addition, several studies have shown that the PI3K/AKT/mTOR pathway is related to the EMT process [31, 32]. AKT can activate EMT, reduce cell–cell adhesion and promote cell metastasis and invasion by down‐regulating E‐cadherin and up‐regulating mesenchymal vimentin [33]. In addition, many studies showed that when the PI3K/AKT‐specific inhibitor LY294002 is added, the rate of cell transition to EMT is reduced, which supports that the PI3K/AKT pathway is associated with EMT. Therefore, based on the importance of the PI3K/AKT pathway in the development of OS and its relevance to EMT, we chose the PI3K/AKT pathway as the study object. As our results showed, after inhibition of PDGFRβ, total PI3K, AKT and mTOR remain unchanged, whereas the phosphorylation levels of PI3K, AKT and mTOR were significantly reduced. This indicates that inhibition of PDGFRβ has an effect on the PI3K/AKT/mTOR pathway. When HOS cells were stimulated with PDGF‐BB, the results were reversed. The phosphorylation level of the relevant factor was increased and the longer the stimulation time, the more obvious that the phosphorylation level was increased. When PDGFRβ binds to its ligand, PDGFRβ‐specific tyrosine kinase is activated to autophosphorylate, thereby activating the PI3K/AKT/mTOR pathway. Based on the earlier results, it is speculated that PDGFRβ may promote the phosphorylation of PI3K and Akt in HOS cells, activate the PI3K/AKT/mTOR pathway and then regulate the changes of EMT‐related proteins, and eventually cause EMT to increase HOS cell migration and invasion. Further research is needed about the mechanism between the PI3K/AKT/mTOR pathway and EMT.

Altogether, this study demonstrates that PDGFRβ plays an important role in invasion and migration of HOS cells. The possible mechanism is by influencing the PI3K/Akt/mTOR pathway and EMT. PDGFRβ can be used as a potential therapeutic target for OS.

## Conflict of interest

The authors declare no conflict of interest.

## Author contributions

SX is responsible for the preliminary design experiment, completion of the experiment, subsequent data analysis work and article writing tasks. HT and JG are mainly responsible for the extraction of RNA. CW, XL, FY and GL are mainly responsible for providing experimental technical guidance. XW is mainly responsible for guiding the ideas of the subject, experimental plans and revision of papers.

## Data Availability

The raw data are available from the corresponding author upon reasonable request.
